# scNovel: a scalable deep learning-based network for novel rare cell discovery in single-cell transcriptomics

**DOI:** 10.1093/bib/bbae112

**Published:** 2024-03-28

**Authors:** Chuanyang Zheng, Yixuan Wang, Yuqi Cheng, Xuesong Wang, Hongxin Wei, Irwin King, Yu Li

**Affiliations:** Department of Computer Science and Engineering, CUHK, Hong Kong SAR, China; Department of Computer Science and Engineering, CUHK, Hong Kong SAR, China; College of Computing, Georgia Institute of Technology, Atlanta, GA, USA; Department of Computer Science and Engineering, CUHK, Hong Kong SAR, China; MLR Lab, Southern University of Science and Technology; Department of Computer Science and Engineering, CUHK, Hong Kong SAR, China; Department of Computer Science and Engineering, CUHK, Hong Kong SAR, China; The CUHK Shenzhen Research Institute, Hi-Tech Park, Nanshan, Shenzhen 518057, China; Institute for Medical Enginering and Science, Massachusetts Institute of Technology, Cambridge, MA, USA; Wyss Institute for Biologically Inspired Engineering, Harvard University, Boston, MA, USA; Broad Institute of MIT and Harvard, Cambridge, MA, USA

**Keywords:** novel rare cell discovery, single-cell analysis, neural network

## Abstract

Single-cell RNA sequencing has achieved massive success in biological research fields. Discovering novel cell types from single-cell transcriptomics has been demonstrated to be essential in the field of biomedicine, yet is time-consuming and needs prior knowledge. With the unprecedented boom in cell atlases, auto-annotation tools have become more prevalent due to their speed, accuracy and user-friendly features. However, existing tools have mostly focused on general cell-type annotation and have not adequately addressed the challenge of discovering novel rare cell types. In this work, we introduce scNovel, a powerful deep learning-based neural network that specifically focuses on novel rare cell discovery. By testing our model on diverse datasets with different scales, protocols and degrees of imbalance, we demonstrate that scNovel significantly outperforms previous state-of-the-art novel cell detection models, reaching the most AUROC performance(the only one method whose averaged AUROC results are above 94%, up to 16.26% more comparing to the second-best method). We validate scNovel’s performance on a million-scale dataset to illustrate the scalability of scNovel further. Applying scNovel on a clinical COVID-19 dataset, three potential novel subtypes of Macrophages are identified, where the COVID-related differential genes are also detected to have consistent expression patterns through deeper analysis. We believe that our proposed pipeline will be an important tool for high-throughput clinical data in a wide range of applications.

## INTRODUCTION

Single-cell RNA sequencing (scRNA-seq), established by Tang et al. in 2009 [1], rapidly becomes popular and important in various biological research field analyses. As a powerful method to profile cell-specific level transcriptome, scRNA-seq makes it possible to analyze individual cells and provide insight into cell heterogeneity and has been widely used in organismal atlases generation [2–6], COVID analysis [7–9], cancer research [10, 11], developmental biology research [12], and so on.

Cell type-specific analysis is a crucial topic in single-cell research and is traditionally carried out by grouping the cell points and then labeling them with marker genes [13–15]. Auto-annotation tools are taking over as a result of the unheard-of surge in the production of cell atlases and their advantages in terms of speed, accuracy, and user-friendliness [16]. In real-world single-cell analysis scenarios, the query dataset may contain novel cell types unseen in the reference set, and the distributions of the reference set and query set usually differ significantly. Hence, it is essential for a tool to not only precisely categorize cell types for query cells but also identify novel cell types that are not present in the reference dataset. Studies have shown the importance of discovering novel rare cells in the fields of biology and medicine. For instance, malignant tumors consist of a minority group of specific cancer stem cells (usually less than 1–2% of the total tumor cells based on the expression of certain cell surface markers) that possess a significant proliferative capacity [17]. Thus, the capability of identifying novel rare cells in individual patients could lead to more personalized treatment approaches based on their unique cellular makeup [18, 19]. However, most prior auto-annotation research concentrated on generic cell-type annotation [20–24] and paid little attention to novel rare cell discovery.

There have been previous endeavors in the field of novel cell discovery. scmap-cluster performs a nearest neighbor search to identify potential matches between cells from different datasets, and uses this information to construct a graph that captures the relationships between cells and clusters them based on their similarity [25]. Similarly, scmap-cell uses an approximate nearest neighbor search method to efficiently find the K-nearest neighbors between the reference and query datasets, which helps reduce the computational complexity and improve the scalability of the algorithm when dealing with large datasets [25]. scPred adopted a random forest classifier to predict cell-type labels, and incorporates unbiased feature selection and dimensionality reduction techniques to improve the accuracy and interpretability of the model [26]. Through routine normalization, discriminative component analysis and learned transformation matrix, scLearn [27] achieves the best performance on novel class detection, compared to the other previous methods. The major limitation of previous work is no one focused on novel rare cell detection and mainly utilized principal component analysis or discriminative component analysis for feature extraction so that the generated novel rare cell feature may lose information [28]. The previous SOTA model scLearn even adds a method named rare cell-type filtering to remove rare cells from datasets.

In this paper, we propose an integrated neural network framework, scNovel, that leverages barcode preprocessing for the auto-detection task. We develop two optional query stage options: the parallel query stage and the sequential query stage. Accordingly, barcode preprocessing is utilized to boost model performance via four modes of novel cell detection. To demonstrate the performance of novel rare cell detection performance of our method, we validate scNovel on various real datasets with different scales, different protocols, and different imbalance degrees. scNovel significantly outperforms previous state-of-the-art novel cell detection models, achieving the highest AUROC performance with statistical significance. scNovel is the only method whose averaged AUROC results are above 94%, up to 16.26% more compared to the second-best one. Furthermore, the scalability of scNovel is demonstrated through the robustness and efficiency across different scales of datasets. It is worth noticing that our work is the first and only novel rare cell discovery tool validated on a million-level atlas [29]. We further apply scNovel on a clinical COVID-19 dataset, discover three potential novel subtypes of Macrophages and conduct a deeper analysis of the disease-related genes’ expression pattern.

## METHODS

### A scalable neural nNetwork for novel rare cell-type discovery

**Figure 1:**
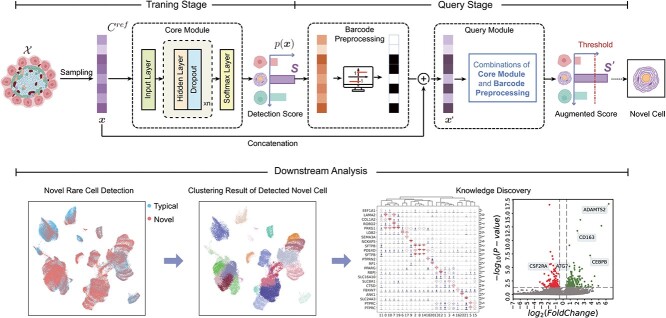
Overview of scNovel. The input $\boldsymbol{x}$ is a single-cell gene expression. We employ a neural network to extract the single-cell feature. Then, map it $C^{ref}$ dimension to get cell-type probability prediction $p( \boldsymbol{x})$. During the training stage, we employ the cross-entropy loss to optimize the network. We introduce two optional query stages, where barcode preprocessing is utilized to augment the predictive capabilities of scNovel. During the query stage, if $p(x)$ is smaller than the threshold, then the corresponding cells are determined as novel. In addition to separating novel cells from typical ones, scNovel could be extended to downstream analysis, including novel rare cell clusters detection and gene analysis.

**Appendix Figure 1:**
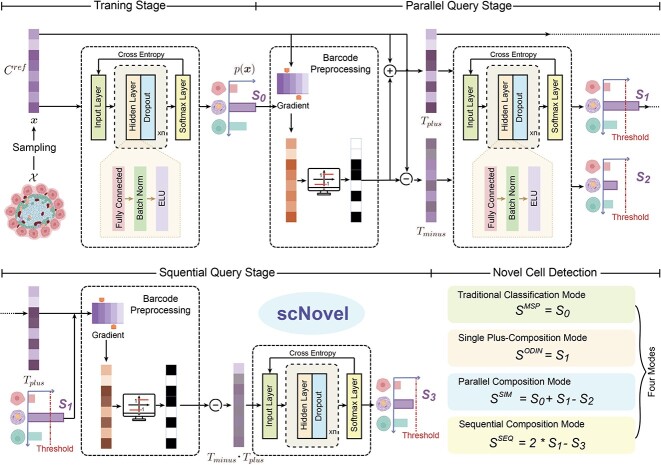
Detailed Framework of scNovel. The input $\boldsymbol{x}$ is a single-cell gene expression. We employ a neural network to extract the single-cell feature. Then, map it $C^{ref}$ dimension to get cell-type probability prediction $p( \boldsymbol{x})$. During the training stage, we employ the cross-entropy loss to optimize the network. We introduce two optional query stages, where barcode preprocessing is utilized to augment the predictive capabilities of scNovel. During the parallel query stage, $T_{plus}(x)$ and $T_{minus}(x)$ are obtained through different operations that composite the input $\boldsymbol{x}$ and the processed barcode data to one vector. Then, at the sequential query stage, $T_{minus}(T_{plus}(x))$ is leveraged for further refinement of the barcode data. We define four modes of novel cell detection based on the proposed query stages.

The basic architecture of our propsoed scNovel is a deep-neural-network-based classifier. As shown in Figure 1, scNovel takes single-cell gene expression as input and outputs the novel cell detection result based on the classifier cell-type probability and the threshold $\lambda $. There are two stages of using scNovel. During the training stage, we adopt cross-entropy loss to train the model to output cell types’ probability prediction after the classifier. During the query stage, if $p(x)$ is smaller than the threshold, then the corresponding cells are determined as novel. To assess the novelty of a given cell type, we design four novel cell detection modes according to the selected query stage option, namely traditional classification mode, single plus-composition mode, parallel composition mode, as well as sequential composition mode (refer to more details in Appendix Figure 1. A thorough explanation of the model framework and training strategy can be found in Appendix C. Once the considered score is over the threshold $\lambda $ (usually defined as 0.5), we ascertain it to be novel. Through the conduction of comprehensive experiments, the predictive power of scNovel is promising even if the cell number and proportion are very small (17 cells out of a million cells). We use AUROC as our main evaluation metric while adopting AUPR and FPR95 metrics for additional reference. Please consult Appendix B for a detailed description of the evaluation metrics.

## RESULTS

### Comprehensive assessment of the detection performance of scNovel

In this section, we compare the performance of our method against existing cell-type classification tools, including scmap-cell and scmap-cluster [25], scPred [26] and scLearn [27]. To ensure that our benchmark comparison is conducted under equitable experimental conditions, we employed a standardized preprocessing procedure for all the benchmark datasets and maintained the default values of all parameters for each method. We considered datasets from diverse imbalance degrees, diverse cell numbers, as well as diverse sequencing platforms to guarantee a more comprehensive comparison. Detailed re-implement and datasets’ information can be found in Appendices A and C.6. As detailed in Appendix Table 1, our selection of datasets encompasses a wide range of widely utilized single-cell sequencing technologies, including 10Xv2 and 10Xv3, and so on. This broad spectrum of technologies was deliberately chosen to provide a robust validation of our method across different platforms, ensuring its generalizability and reliability in various single-cell sequencing contexts.

**Figure 2:**
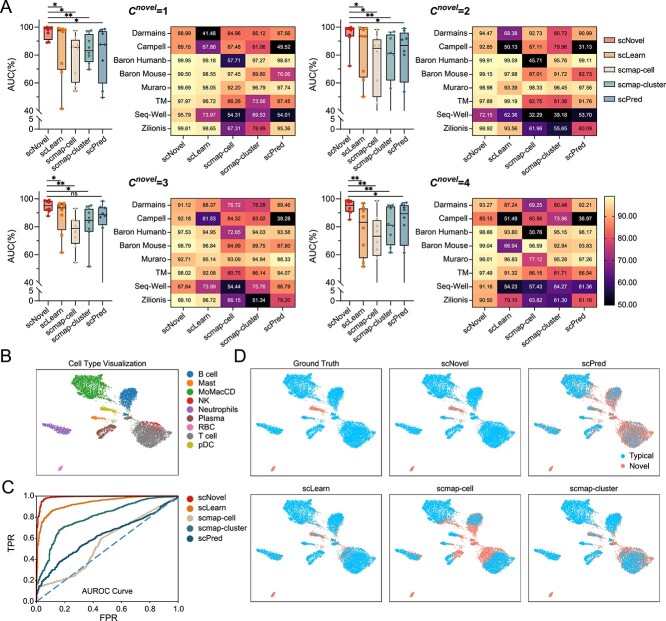
(**A**) AUROCs of different methods with various $C^{novel}$. The box plots show that scNovel has higher mean but smaller interquartile range of AUROCs than all the other methods, showing a stable novel rare cell detection ability among various datasets and $C^{novel}$. *P*-value with notation * means *P*¡0.05, with notation $ns$ means no significance. The heatmaps show the detailed AUROC values of different methods and datasets. (**B**) Cell-type UMAP visualization of Zilionis dataset. (**C**) The AUROC curves of various methods are evaluated, with scNovel demonstrating superior TPR over all other methods, regardless of the FPR. (**D**) UMAP visualization of the novel cell discovery task on the Zilionis dataset with $C^{novel}$=2. The result of scNovel is the closest to the ground truth, while the other ones have too many false positive predictions. scNovel outperforms other methods in detecting novel rare cell types, uniquely identifying pDC and RBC. Unlike scLearn, scPred, scmap-cell and scmap-cluster, which inaccurately annotate T cells and Neutrophils, scNovel achieves the most precise detection across all cell types, successfully pinpointing RBC and pDC.

As shown in Appendix Table 2, compared with previous models, our proposed scNovel significantly improves the averaged performance via having the lowest FPR95 score, the highest AUROC and the highest AUPR scores on the novel rare cells discovery task among different datasets. Detailed description of the above-mentioned evaluation metrics can be found in the Appendix B. Given our emphasis on the detection of novel and rare cells, our initial evaluation of scNovel concentrates on its performance with a limited set of novel cell classes that are absent from the training data. In this context, we use the notation $C^{novel}$ to represent the number of novel classes present in the test set. From the results presented in Appendix Table 2, it is noticeable that there is a decreasing trend in performance as the number of novel classes ($C^{novel}$) increases. This indicates that the task becomes progressively more challenging as the number of novel classes in the test set increases. Namely, scNovel achieves a 96.33% AUROC when $C^{novel}$ is equal to 1, surpassing the next best performing method, scLearn, by a substantial margin of 12.33% AUROC. Across all $C^{novel}$, our scNovel gains on an average 11.75% AUROC more compared to the second best method scLearn, demonstrating that scNovel is robust for different $C^{novel}$, and is outstanding compared to other models. The box plots in Figure 2(A) further demonstrate the robustness of scNovel by showing not only higher AUROC values but also smaller interquartile ranges across various dataset sizes ($\sim $ 1k cells to $\sim $ 60k cells) and different protocols. The statistical analysis, using a one-tailed *t*-test, consistently demonstrates that scNovel outperforms other methods with significant statistical significance in most scenarios. This indicates that the adopted neural network shows robust performance for single-cell sequencing feature extraction and works well for novel rare-cell feature extraction. The cell-type distribution and detection results are visualized in Figure 2(B).

We also show the AUROC curves in Figure 2(C). for the Zilionis dataset with $C^{novel}=2$. According to the AUROC curve, we could find that scNovel not only dominates in AUROC space [30] but also achieves superior true positive rate (TPR) over all other methods, regardless of the FPR. As shown in Figure 2(B and D), Notably, scNovel has the best novel rare cell detection performance and is the only method that can detect the two novel rare cell types, including plasmacytoid dendritic cell (pDC) and red blood cell (RBC). Compared with the ground truth label, scLearn, scPred, scmap-cell and scmap-cluster give more incorrect annotations on the T cell and Neutrophils than scNovel. scPred even almost fails on this task, as it detects the majority of cells to be novel. In contrast, scNovel demonstrates the most accurate novel rare cell detection result on all cell types and successfully finds the two novel rare cells, RBC and pDC.

### Consistent performance with various C^novel^ in different modes

In this section, we investigate our model performance with various novel cell class numbers $C^{novel}$ in different modes. We begin with showing our model performance with four different query modes:

-
$S^{MSP}$ in Equation (C6), which uses the maximum prediction probability as score;
-
$S^{ODIN}$ in Equation (C10), which utilizes only $T_{minus}$ barcode preprocessing;
-
$S^{SEQ}$ in Equation (C13), which sequentially utilizes $T_{minus}$ and $T_{plus}$ barcode preprocessing;
-
$S^{SIM}$ in Equation (C12), which simultaneously employs $T_{minus}$ and $T_{plus}$ barcode preprocessing.

**Figure 3:**
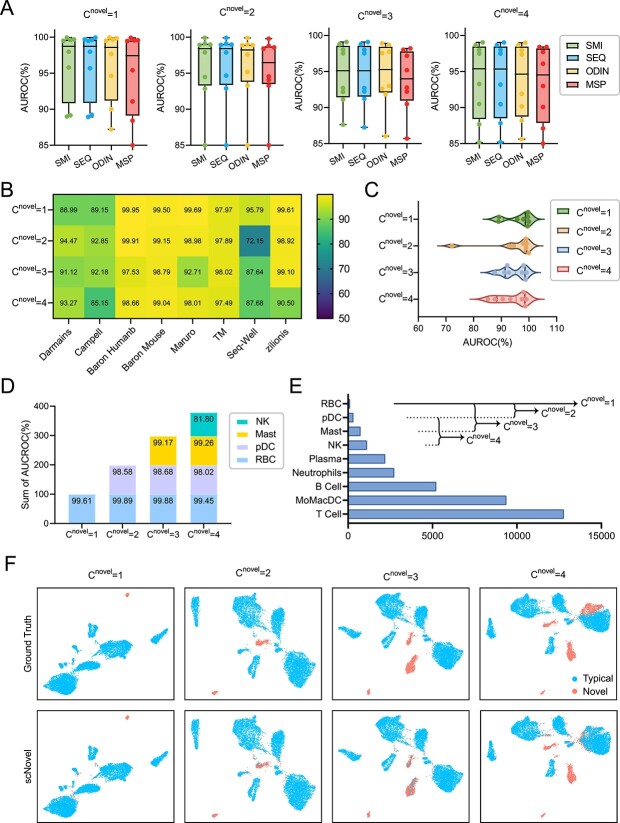
(**A**) The box plots of AUROCs of different novel cell detection modes with $C^{novel}$ ranging from 1 to 4. (**B**) Heatmap of the detailed AUROC values of $S^{SIM}$ mode on all the considered datasets. (**C**) The violin plot of the $S^{SIM}$ mode of scNovel’s AUROCs with different novel class numbers $C^{novel}$ on various datasets. We could find that the performance of scNovel is stable with various $C^{novel}$. (**D**) The accumulation bar plot of AUROC values of each novel rare cell detected from the Zillion dataset with $C^{novel}$ ranging from 1 to 4. (**E**) Taking the Zillion dataset as an example, the bar plot shows the experimental setting with different $C^{novel}$. (**F**) The UMAP visualization comparison between scNovel and the ground truth of the novel cell discovery task on the Zilionis dataset with different $C^{novel}$.

Through the detailed ablation study (Appendix Table 4 and Figure 3A), we come to the conclusion that $S^{SIM}$ should be the best mode for novel rare cell-type discovery tasks. Therefore, future efforts will focus on devising more sophisticated methods, potentially exploring various forms of sharpness metrics (as shown in Equations C12 and C13), to enhance the distinction between typical and novel cells, thereby further elevating the model’s performance. We then investigate scNovel’s performance with different $C^{novel}$ of $S^{SIM}$ mode in Figure 3(B and C) in detail. Overall, scNovel has the best average performance across all datasets, as well as has the smallest variance (Figure 3C) when $C^{novel}$ is 1. To be specific, scNovel gets 22.82% FPR95, 96.33% AUROC and 99.76% AUPR when the model is only required to predict one novel cell type. Detailed AUROC performance on each dataset is shown in Figure 3(B). Intuitively, as $C^{novel}$ increases, the novel cell distribution $\mathcal{P}_{novel}$ becomes more and more complicated. As a consequence, the AUROCs of $C^{novel}$=2, 3 and 4 decrease to 94.29, 94.6 and 94.16%, respectively. Figure 3(D) shows the accumulated AUROCs of each novel cell type of dataset Zillion with various $C^{novel}$. Figure 3(E) shows the corresponding data-split settings of the four $C^{novel}$. With the increase of $C^{novel}$, the AUROCs of RBC are stable. This also suggests that the novel rare cell detection performance of scNovel is mainly affected by the cell type, and it is robust to the various $C^{novel}$. In Figure 3(F), we have presented a comparative analysis of UMAP visualizations for scNovel, utilizing a threshold of 0.5, against the ground truth for the task of novel cell discovery within the Zilionis dataset. This comparison indicates that our proposed scNovel method achieves consistently meaningful visualization performance across different values of $C^{novel}$, employing a default threshold of 0.5.

### Superiority in rare population identification under batch effects

**Figure 4:**
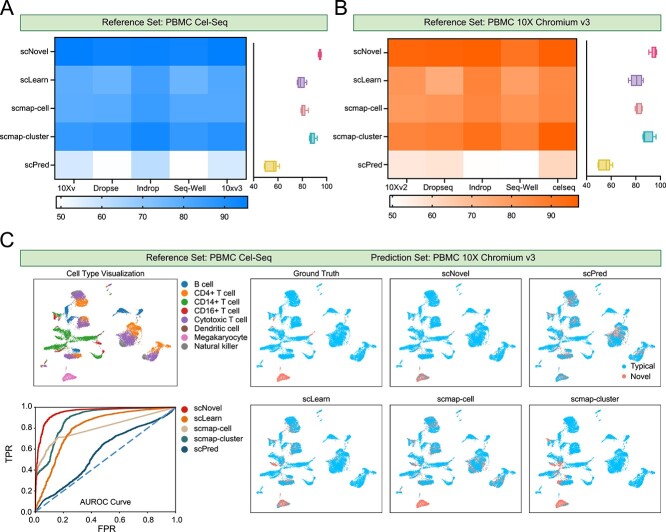
(**A**) Overall novel rare cell detection AUROCs on the datasets generated by different protocols, with reference dataset PBMC Cel-Seq. (**B**) Overall novel rare cell detection AUROCs on the datasets generated by different protocols, with the reference dataset PBMC 10X Chromium v3. (**C**) The UMAP visualization of ground truth cell-type, AUROC curves and the UMAP visualization of novel cell discovery task of various methods. All methods are trained on PBMC Cel-Seq, and tested on PBMC 10X Chromium v3. Only scNovel and scLearn detect all Megakaryocyte cells, while scNovel has few false positive predictions than scLearn.

In the real application scenario, training and applying the rare cell detection tools can be conducted on different dataset protocols and platforms with heavy batch effects. These systematic variations are crucial, particularly when analyzing rare cell populations, and must be carefully addressed to ensure accurate and reliable results. Therefore, as shown in Figure 4(A and B), we collect PBMCbench datasets from 10Xv2, 10Xv3, Drop-Seq, Cel-Seq, InDrop and Seq-Well are considered to evaluate scNovel’s ability to detect novel rare cells across platforms

scLearn, which is the second-best model in intra-platform experiments, is shown to suffer from the effect of batch effects, achieving only 78.95% for Cel-Seq reference dataset (Figure 4A) and 80.61% for 10Xv3 reference dataset (Figure 4B). Our proposed scNovel outperforms scmap-cluster by 6% and scLearn by 16%, suggesting that scNovel is robust to the potential obstacles in cross-platform annotation tasks. To further investigate the performance of model performance under batch effect, we show the UMAP visualization result and AUROC results in Figure 4(C), with Cel-Seq as reference set and test on 10Xv3 set query set. There are two novel cell types, including Dentritic cells and Megakaryocyte. Only scNovel and scLearn successfully detect all Megakaryocyte cells, while scmap-cell, scmap-cluster, and scPred miss a part of Megakaryocyte cells. Compared with scLearn, our proposed scNovel has even fewer false positive predictions, while scLearn wrongly detects CD16+ monocyte and Cytotoxic T Cell to be novel. Also, according to the AUROC curve, we could find that our proposed scNovel has a better TPR than all other models, regardless of the FPR. This proves that our proposed scNovel upholds its excellent performance in the novel rare cell-type discovery tasks under the impact of systematic variations.

### Enhanced scalability of scNovel through robust processing efficiency in million-level dataset

**Figure 5:**
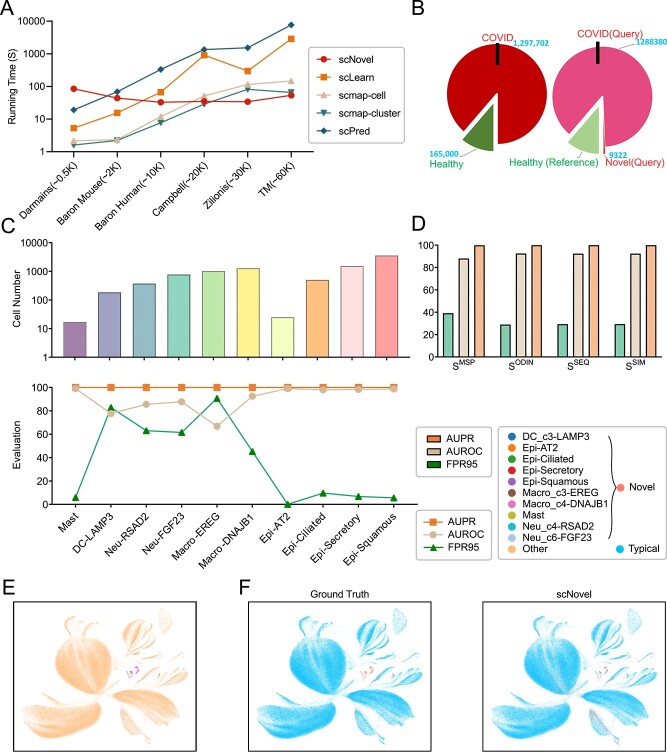
(**A**) Time complexity analysis of different methods on datasets with different scales. scNovel exhibits exceptional scalability, requiring the least amount of time even when processing TM dataset that scales up to 60k cells. (**B**) Experiment setting of COVID Altas. (**C**) The $\mathcal{D}_{query}^{novel}$ cell number statistic and each cell-type detection results. The $\mathcal{D}_{query}^{novel}$ has 10 cell types and total of 9332 cells. We could find that the novel rare cell detection results are not affected by the cell number. The mast, the rarest cell, is detected better than Macro-EREG, whose cell number is large. (**D**) Results of novel rare cell detection with different score functions. (**E**) Cell-type UMAP visualization of the considered COVID-19 immune atlas. (**F**) UMAP visualization of the novel cell discovery task of scNovel on the COVID-19 immune atlas with $C^{novel}$ = 10.

In the context of real-world single-cell analysis, computational efficiency is of vital importance and is viewed as the main impediment to achieving scalability. From Figure 5A, the running time of scNovel remains relatively consistent across datasets with varying samples. For the datasets with more than 30k cells, scNovel reduces the runtime by up to 25% compared to the fastest of our four comparison models, scmap-cluster. On the analysis of the largest dataset, TM, scNovel provides a computation speed over 120 times faster than scPred, showing that our proposed method has the potential to facilitate rapid novel cell detection of large-scale datasets. Our analysis shows the model’s running time is mainly influenced by two factors: computation and weight updates, and Pytorch Dataloader preparation, which remains constant across datasets. However, smaller datasets require more frequent Dataloader preparations, leading to longer running times, as seen with the Darmanis dataset compared to the larger Zilionis dataset. Hence, dataset size directly affects the model’s overall running time. The significance of scalability for detection tools becomes more pronounced as the size of the cell atlas increases.

To further validate the robustness of our performance on a large, imbalanced dataset, we apply scNovel to a million-scale scRNA-seq dataset COVID-19 immune atlas [29](1 462 702 cells). The scRNA-seq dataset COVID-19 immune atlas has 64 cell types, divided into three groups, including healthy, moderate (COVID) and severe(COVID). The healthy group has 54 cell types,while 10 cell types only appear in COVID groups (shown in Figure 5B), and the 10 cell types are selected as novel cell types. We further elaborate on the impact of the 10 specific cell types on our experimental outcomes, as illustrated in the results presented in Figure 5(C). Shown in Figure 5(D), the performance of $S^{MSP}$ mode is lower than that of $S^{ODIN}$ mode, $S^{SEQ}$ mode and $S^{SIM}$ mode, which suggests the importance of barcode preprocessing. Figure 5(C) shows that the Epi cell types, including Epi-Squamous, Epi-Secretory, Epi-Ciliated, and Epi-AT2, have low FPR95, high AUROC, and high AUPR. The underlying reason is that Epi cell types only appear in $\mathcal{D}_{query}^{novel}$ so that it is easier for our model to detect them. For the same reason, the Mast, which only has 17 cells, has the same high AUROC. In contrast, the performance is relatively lower in Macro-DNAJB1, Macro-EREG, Neu-FGF23, Neu-RSAD2 and DC-LAMP3. For Macro-DNAJB1 and Macro-EREG, there are other subgroup cells of Macro in $\mathcal{D}_{ref}$, such as Macro-C1QC and Macro-CCL3L1. For Neu-FGF23 and Neu-RSAD2, there are Neu-IL1B and Neu-CST7 in $\mathcal{D}_{ref}$. For DC-LAMP3, the $\mathcal{D}_{ref}$ contains DC-CLEC9A and DC-LILRA4. We could notice that the novel rare cell detection performance is not related to the cell numbers. The mast, the rarest cell, is detected better than Macro-EREG, whose cell number is large. As shown in Figure 5(E) and (F), scNovel is capable of accurately identifying 10 novel rare cell types that are exclusively present in COVID-19 cases within datasets comprising millions of cells. This achievement underscores the scalability of scNovel and highlights its potential for deployment in large-scale real-world applications.

### Potential novel subtypes discovery and functional investigation in bronchoalveolar immune cell atlas in COVID patient

Studies have shown the importance of cell-type-specific analysis of the genetic heterogeneity of specific cell types in the field of biomedicine [31]. For instance, COVID single-cell atlas [29] detects 64 cell types further to analyze the relationship between cell gene expression and diseases. However, the detection process of novel cell types is time-consuming and needs prior knowledge. Our proposed scNovel could find novel rare cells automatically: (1) detect the novel cells of the query set; (2) employ Leiden clustering to generate cell clusters; and (3) utilize the novel cell clusters for further analysis.

**Figure 6:**
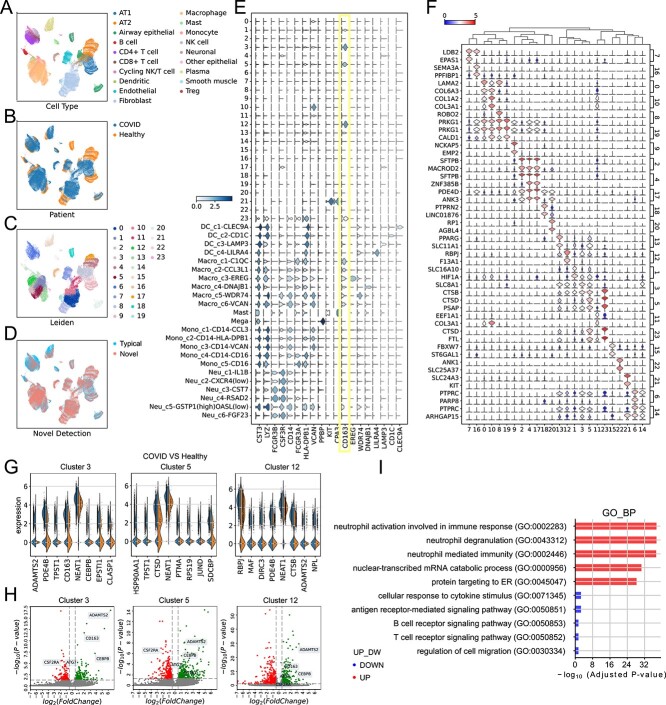
(**A**) UMAP visualization of each cell type in the query dataset. There are a total of 19 cell types. (**B**) UMAP visualization of patient distribution. (**C**) The Leiden clustering results of detected novel cells. In total, there are 24 new cell types. (**D**) UMAP visualization of the novel cell detection result via scNovel with threshold 0.45. (**E**) The stacked violin plot overview of the gene expression in typical cells of reference set and new cell types. According to the gene expression, the new cell clusters are different from the typical cells in the reference set. (**F**) The stacked dot plot overview of the top-important marker genes expression for each cell type. (**G**) The comparison of the expression level between the top 8 biomarkers in the COVID group and the rest of the patients. (**H**) The Volcano figure of cluster 3, cluster 5 and cluster 12. (**I**) GO analysis in novel Macrophages between COVID samples and Healthy Samples.

In this study, with a 1.5-million-scale dataset as the reference set, we apply the above analysis pipeline to a published COVID-19 dataset [32] (GEO accession number GSE171524), containing 116 314 cells collected from 7 healthy controls and 20 COVID-19 samples. We show the UMAP visualization of cell-type annotation (Figure 6A) and patient annotation (Figure 6B). Figure 6C presents the novel cell detection result. In our setting, B cell and T cell are present in the reference set, while AT1 and AT2 are left for testing. Figure 6D, as detected by scNovel, illustrates the distributions of typical and novel cells, demonstrating the tool’s effectiveness in identifying and differentiating between cell types. Combining the results in Figure 6C and Figure 6D, we can find that B cell and T cell are less likely to be predicted to be novel, but AT1 and AT2 are detected as novel cells successfully. To further ensure the discovered novel cells are different from the cells in the reference set, we present the gene expression in Figure 6E. It directly supports that the newly discovered cells are different from the cells in the reference set. We also carry out analysis on the gene differentiation of different novel cell clusters by showing the highest gene expression of each novel cell cluster and their relation in Figure 6F. We find that clusters 3, 5, and 12 have high CD163 expression, which means that they may be subtypes of Macrophage. Then, we conduct deeper analyses of the selected three clusters to study the relationship between their gene expression and COVID-19. We show the expression level between the top 8 biomarkers in each cluster (Figure 6G). From the volcano figure in Figure 6H, seven genes have higher expression in COVID patients, and some are already supported by past research, including ADAMTS2 [33], CD163 [34, 35], CEBPB [36], CTSD, LDLRAD3, NEAT1 [37] and TPST1. Moreover, nine genes have lower expression in COVID patients, which are also supported by the existing works, including ATG77 [38], CSF2RA [39], DST, FARP1, FRMD4A, HLA-DPB1, HLA-DRB1, RASAL2 and TFRC. Rather than only analyzing differential gene expression in a single cluster, selecting biomarkers that satisfy all three criteria in the dependent clusters makes the analysis results more reliable. Finally, we conducted a Gene Ontology (GO) analysis in Figure 6I, supplemented by gene set enrichment analysis, to investigate changes in biological pathways between macrophages with differing COVID statuses. Our results indicate significant upregulation in COVID samples, particularly in neutrophil activation related to immune response, neutrophil degranulation, and neutrophil-mediated immunity. By applying scNovel to a COVID-19 dataset, we have demonstrated its effectiveness in accurately identifying unique cell types. This success suggests the potential of scNovel as a critical tool in accelerating diagnostics and response in future pandemics or diseases.

## DISCUSSION

The development of scRNA-seq methods has increased the demand for single-cell analysis tools. Previous research works mainly focus on cell-type annotation, but pay little attention to novel rare cell discovery task, which is important for classifier’s reliability and cell discovery. To the best of our knowledge, we are the first one focusing on novel rare cell discovery, and we propose the first corresponding auto-detection model named scNovel, an open-source Python package that integrates neural network and barcode preprocessing. With extensive experiments on several scRNA-seq datasets with different scales, different generation protocols and different $C^{novel}$, we have demonstrated an effective novel rare cell discovery ability. We have also shown the fast running speed of scNovel on datasets with different scales, which proves the scalability. Moreover, we evaluate our model on a million-level dataset, which further shown the scalability and novel rare cell discovery capacity. Also, we employ the scNovel to detect novel rare cell clusters for more trustable disease gene analysis.

In future work, we have several suggestions for scNovel improvement. Firstly, a better score function could improve scNovel performance. Specifically, we could utilize up-sharpness and down-sharpness multiple times. Secondly, we could employ more biology information. For instance, we leverage both scRNA-Seq and single-cell chromatin accessibility sequencing for novel rare cell discovery. In all, we believe that scNovel is an effective and efficient tool for single-cell analysis, especially its ability and scalability of rare cell types.

**Key Points**

- scNovel is a scalable deep learning-based network for novel rare cell discovery in single-cell transcriptomics, and the first method to use the barcode preprocessing on novel rare cell detection on the scRNA-seq data.
- We apply scNovel on extensive intra-dataset and inter-protocol novel rare cell detection experiments, even considering a large, imbalanced million-scale dataset. Notably, it achieves an average AUROC performance exceeding 94%, surpassing the second-best method by up to 16.26%.
- Within the context of the profoundly imbalanced clinical COVID-19 dataset, a comprehensive analysis has unveiled the existence of three promising novel subtypes of macrophages.
- We developed a pipeline for the identification of novel rare cells and the analysis of differential gene expression. Our proposed scNovel-based analytical framework extends beyond its application to COVID-19, as it can be seamlessly tailored to investigate genes associated with various other diseases.

## Data Availability

No new data was generated from this study. All data used in this study is publicly available as shown in Appendix Table 1.

## CODE AVAILABILITY

scNovel is available as an independent python package at https://github.com/chuanyang-Zheng/scNovel.

## A. Datasets and preprocessings

In this work, we considered a total of 15 single-cell expression datasets from small scale ($\sim $1k cells) to large scale ($\sim $1.5m cells). Detailed information on cell numbers, class numbers, sequencing protocols and sources of each dataset is shown in Appendix Table 1. We incorporated datasets with different complexity and sequencing protocols to show the effectiveness and generalization of our proposed scNovel. We obtain the corresponding cell-type labels from their original paper to achieve the learning in a supervised manner.

All of these labeled single-cell profiles went through the same preprocessing procedure to achieve more precise prediction results. To mitigate the impact of outlier genes, log transformation and normalization steps were implemented following the standard preprocessing pipeline outlined in the Scanpy [21] tutorial, where the scale parameter was manually adjusted in the normalization function. We then split all the datasets using the following steps: (1) select the single-cell sequences of the top $C^{novel} \in \mathbb{N}^{+}$ most rare cell types from the dataset and utilize them to build $\mathcal{D}_{query}^{novel}$; (2) utilize 75% of the remaining data as $\mathcal{D}_{ref}$, and the other 25% of the remaining data as $\mathcal{D}_{query}^{typical}$; and (3) the $\mathcal{D}_{ref}$ is employed for the reference set, while $\mathcal{D}_{query}^{typical}$ and $\mathcal{D}_{query}^{novel}$ are combined to build query set $\mathcal{D}_{query}$.

## B. Evaluation metrics

We adopt the following three metrics to measure the performance of different models on the novel rare cell discovery task. A better model should have lower FPR95, higher AUROC and higher AUPR, following the previous works [26, 47]. The AUROC is the most important evaluation metric as our dataset is imbalanced:

(1) **FPR95**: FPR at 95% TPR is the probability that a negative sample (novel sample) is misclassified as a positive sample (typical sample) when the TPR is 95%.
(2) **AUROC**: AUROC is the Area Under the Receiver Operating Characteristic curve, which is a threshold independent metric [30]. The AUROC shows the relationship between TPR and FPR. It could be interpreted as the probability that a positive sample has a higher score than a negative sample [48].
(3) **AUPR**: AUPR is the Area Under the Precision-Recall curve, which is also a threshold independent metric [49, 50]. It depicts the relationship between precision =$\frac{TP}{TP+FP}$and recall=$\frac{TP}{TP+FN}$. The AUPR is high in our experiments because the amount of novel cells is small compared to typical cells.

## C. Appendix methods

### C.1 Neural network for rare cell feature extraction

To better extract input features, we employ a neural network classifier. The single-cell transcriptome data are processed by a neural network classifier $f$ to get prediction probability $p(\boldsymbol{x})$. The classifier has several hidden layers, whose structure is shown in Appendix Figure 1. Besides a Fully-Connected Layer (FC) in each hidden layer, we utilize batch normalization (BN), Exponential Linear Unit (ELU) activation function and dropout sequentially to speed up training, avoid overfitting and elevate predictive performance. Our network design encompasses architecture, activation functions, and loss functions that are consistent with methodologies applied in similar studies focusing on rare cells and classes with limited quantities [16, 51, 52]. This alignment underscores our commitment to precisely handling rare cells to enhance feature extraction and improve performance:

(C.1)
\begin{align*}& \begin{split} \boldsymbol{x}^{l-1}_{BN}&=BN(\boldsymbol{x}^{l-1}),\\ \boldsymbol{x}^{l-1}_{ELU}&=ELU(\boldsymbol{x}^{l-1}_{BN}),\\ \boldsymbol{x}^{l-1}_{Dropout}&=Dropout(\boldsymbol{x}^{l-1}_{ELU}), \end{split}\end{align*}

where $\boldsymbol{x}^{l-1}$ is the feature output of the $(l-1)$th FC layer; ELU processes features with Equation (C.2)

(C.2)
\begin{align*}& ELU(\boldsymbol{x})=\left\{ \begin{array}{@{}lr} x,\quad if\ \boldsymbol{x} \geq 0, &\\ \alpha e(e^{\boldsymbol{x}}-1),\quad if\ \boldsymbol{x} < 0. &\\ \end{array} \right.\end{align*}

**Appendix Table 1:** Datasets considered

Datasets	Cell numbers	Class numbers	Protocols	Source
Darmains	466	9	SMARTer	Darmains *et al*. [40]
Muraro	2122	9	Cel-Seq2	Muraro *et al*. [41]
Baron Mouse	1886	13	inDrop	Baron *et al*. [42]
Baron Human	8569	14	inDrop	Baron *et al*. [42]
Seq-Well	18 966	7	Seq-Well	Ding *et al*. [43]
Campbell	21 086	35	Drop-Seq	Campbell *et al*. [44]
Zilionis	34 558	9	inDrop	Zilionis *et al*. [45]
TM	54 865	55	10X Genomics	Schaum *et al*. [46]
PbmcBench 10X (V2)	23 154	9	10X Genomics (v2)	Ding *et al*. [43]
PbmcBench 10X (V3)	19 690	8	10X Genomics (v3)	Ding *et al*. [43]
PbmcBench (CEL-Seq)	19 754	7	CEL-Seq2	Ding *et al*. [43]
PbmcBench (Drop-Seq)	23 154	9	Drop-Seq	Ding *et al*. [43]
PbmcBench (inDrop)	21 832	7	inDrop	Ding *et al*. [43]
PKU-Covid Atlas	1 462 702	64	10X Genomics	Ren et al. [29]
GSE171524	116 314	19	10X Genomics	Melms et al. [32]

Given input $\boldsymbol{x}$, the proposed network process is defined as the following:

(C.3)
\begin{align*}& \begin{split} \boldsymbol{x}_{1}&=InputLayer(\boldsymbol{x}),\quad\quad\quad \boldsymbol{x}_{2}=HiddenLayer_{1}(\boldsymbol{x}_{1}),\\ \boldsymbol{x}_{3}&=HiddenLayer_{2}(\boldsymbol{x}_{2}),\quad \boldsymbol{x}_{4}=HiddenLayer_{3}(\boldsymbol{x}_{3}),\\ f(\boldsymbol{x})&=FC(x_{4}),\quad\quad\quad\quad\quad p(\boldsymbol{x})=Softmax(f(\boldsymbol{x})), \end{split}\end{align*}

where $\,\boldsymbol{x}_{1}$ is the feature output after $InputLayer$; $\boldsymbol{x}_{2}$, $\boldsymbol{x}_{3}$ and $\boldsymbol{x}_{4}$ are the features after $HiddenLayer_{1}$, $HiddenLayer_{2}$ and $HiddenLayer_{3}$ respectively; $f(\boldsymbol{x})$, processed by FC, is the feature of input $\boldsymbol{x}$; $p(\boldsymbol{x})$ is the prediction probability. More details can be found in Appendix C.

### C.2 Training stage

scNovel takes a single-cell RNA expression matrix M as an input, where each column corresponds to a gene and each row corresponds to a cell. As shown in Appendix Figure 1, we first consider a supervised multi-class classification task problem. We denote $\mathcal{D}_{ref}=\{\boldsymbol{x_{i}},y_{i}\}_{i=1}^{n^{ref}}$ as the reference set with $C^{ref} \in \mathbb{N}^{+}$ cell types. $\mathcal{X}$ represents the input space, while $\mathcal{Y}=\{1,2...,C^{ref}\}$ signifies the label space with $C^{ref}$ classes. $\mathcal{P}_{typical}$ and $\mathcal{P}_{novel}$ represent the distribution of typical cells and novel cells. Given a dataset, with trainable parameter $\theta \in \mathbb{R}^{p}$, a classifier f: $\mathcal{X} \rightarrow \mathbb{R}^{C^{ref}}$ maps an input to the output space $\mathbb{R}^{C^{ref}}$. Here, the cross-entropy loss is calculated for optimization:

(C.4)
\begin{align*} & p(\boldsymbol{x})=\operatorname{softmax}(f(\boldsymbol{x};\theta),j)=\frac{e^{f_{j}(\boldsymbol{x};\theta)/T}}{\sum_{i=1}^{k}e^{f_{i}(\boldsymbol{x};\theta)/T}},\end{align*}

(C.5)
\begin{align*}& \mathcal{L}_{\mathrm{CE}}(f(x;\theta),y)=-\log p(y\lvert\boldsymbol{x})=-\log p_{y}(\boldsymbol{x}),\end{align*}

where input $\boldsymbol{x}$ is the gene expression profile of a cell, which is a row of single-cell RNA expression matrix M; $f(\boldsymbol{x})$ is the feature of input $\boldsymbol{x}$; $f_{i}(\boldsymbol{x})$ is the $i^{th}$ element of $f(\boldsymbol{x})$, given the label $i$; $p(\boldsymbol{x})$ is the prediction probability of input $\boldsymbol{x}$; $p(y\lvert \boldsymbol{x})$ is $y^{th}$ element of $p(\boldsymbol{x})$, given the ground truth label $y$; T is a hyperparameter, and we use 1000 as the default value (follow the parameter setting in [47]).

In the training stage, we minimize the cross-entropy loss in Equation (C.5) with the reference set $\mathcal{D}_{ref}$. In the query stage, samples from novel distributions $\mathcal{P}_{novel}$ may appear, and the cell-type set of $\mathcal{P}_{novel}$ has no intersection with the reference set label space $\mathcal{Y}$. Such samples come from novel distribution $\mathcal{P}_{novel}$ should be detected by our proposed model.

### C.3 Barcode preprocessing

The novel rare cell discovery task could be formulated as a binary classification problem. We determine an input $\boldsymbol{x} \in \mathcal{X}$ coming from $\mathcal{P}_{typical}$ or $\mathcal{P}_{novel}$ via the process shown in Appendix Figure 1. Given an input, we utilize a classifier to get prediction probability $p(\boldsymbol{x})$. The simplest score function, named traditional classification model, is defined as the following [53]:

(C.6)
\begin{align*}& S^{MSP}(\boldsymbol{x})=\underset{j \in \mathcal{Y}}{max}\ \ p_{j}(\boldsymbol{x}),\end{align*}

where we choose the max probability as the decision score; $p_{j}(\boldsymbol{x})$ is the $j^{th}$ element of $p(\boldsymbol{x})$. Based on this setting, we define the binary decision function to be

(C.7)
\begin{align*}& G(\boldsymbol{x})=\left\{ \begin{array}{@{}lr} Typical,\quad if\ S(\boldsymbol{x}) \geq \lambda, &\\ Novel,\quad if\ S(\boldsymbol{x}) < \lambda. &\\ \end{array} \right.\end{align*}

where $S(*)$ is the score function and $G(*)$ is the novelty classification that $x$ belongs to; $\lambda $ is a hyperparameter that we set as a threshold with a default value of 0.5 (recommended). Usually, samples with a score that reaches the threshold are classified as typical of a certain cell type that the max probability corresponds to, and those with a score below the threshold are classified to be novel. To be more specific, the higher the score is, the lower probability that the cell type is novel.

**Parallel query stage** To further improve the model performance, we add barcode preprocessing when testing on the query dataset. The core idea is that adding input perturbations to the sample could help enlarge the margin between typical cell data and novel cell data [47]. Hence, the barcode preprocessing can have a higher effect on typical cell types (the score will be changed more, including increasing more and decreasing more), and it has a lower effect on novel cell types ( the score will not change too much). As shown in Appendix Figure 1, we design two types of barcode preprocessing $T_{minus}$ and $T_{plus}$ with different composition operations:

(C.8)
\begin{align*} & \begin{aligned} S_{\hat{y}}(\boldsymbol{x})= \underset{j \in \mathcal{Y}}{max}\ p_{j}(\boldsymbol{x}), \end{aligned}\end{align*}

(C.9)
\begin{align*}& \begin{aligned} T_{plus}(\boldsymbol{x})=\boldsymbol{x}-\epsilon sign(-\nabla_{\boldsymbol{x}}\ logS_{\hat{y}}(x) ) = \boldsymbol{x}+\epsilon sign(\nabla_{\boldsymbol{x}}\ logS_{\hat{y}}(x) ),\\ T_{minus}(\boldsymbol{x})=\boldsymbol{x}+\epsilon sign(-\nabla_{\boldsymbol{x}}\ logS_{\hat{y}}(\boldsymbol{x}) ) =\boldsymbol{x}-\epsilon sign(\nabla_{\boldsymbol{x}}\ logS_{\hat{y}}(\boldsymbol{x}) ). \end{aligned}\end{align*}

where $\epsilon $ is the perturbation hyperparameter, whose default value is 0.0014; for the two barcode preprocessing methods, we first compute the gradient via backpropagation, and then the gradient will multiply by hyperparameter $\epsilon $; $T_{minus}(x)$ minus the multiplication result, while $T_{plus}(x)$ plus the multiplication result; usually, $S(T_{minus}(x))$ is larger than S(x), while $S(T_{plus}(x))$ is smaller than $S(x)$. We design Single Plus-Composition Mode, denoted as $S^{ODIN}$, which employs the following equation for barcode preprocessing [47]:

(C.10)
\begin{align*}& S^{ODIN}(\boldsymbol{x})=\underset{j \in \mathcal{Y}}{max}\ \ p_{j}(T_{plus}(\boldsymbol{x})).\end{align*}

In fact, we could write a general formulation of Equation (C.10) as follows:

(C.11)
\begin{align*}& \begin{aligned} S^{ODIN}(\boldsymbol{x})&=\underset{j \in \mathcal{Y}}{max}\ \ \{ \underbrace{p_{j}(\boldsymbol{x})}_{magnitude}+ \underbrace{p_{j}(T_{plus}(\boldsymbol{x}))-p_{j}(\boldsymbol{x})}_{up-sharpness}\}, \end{aligned}\end{align*}

where the magnitude is the score of input $x$ without barcode preprocessing; the up-sharpness is the increment in score after the $T_{plus}$ process. The limitation of $S^{ODIN}$ is that it only considers up-sharpness but does not consider down-sharpness, which is the decrease in score after the $T_{minus}$ process. Hence, we could simultaneously calculate up-sharpness and down-sharpness at the same input $x$, which is $S^{SIM}$, named Parallel Composition Mode defined in the following:

(C.12)
\begin{align*} S^{SIM}(\boldsymbol{x})&=\,\underset{j \in \mathcal{Y}}{max}\{ \underbrace{p_{j}(\boldsymbol{x}) }_{magnitude}+ \underbrace{p_{j}(T_{plus}(\boldsymbol{x}))-p_{j}(\boldsymbol{x})}_{up-sharpness}+\underbrace{p_{j}(\boldsymbol{x})-p_{j}(T_{minus}(\boldsymbol{x}))}_{down-sharpness}\}\nonumber\\ &=\,\underset{j \in \mathcal{Y}}{max}\{ p_{j}(\boldsymbol{x})+p_{j}(T_{plus}(\boldsymbol{x}))-p_{j}(T_{minus}(\boldsymbol{x}))\}. \end{align*}

To summarize, the parallel query stage considers two novel cell detection modes, namely Single Plus-Composition Mode and Parallel Composition Mode. Score functions $S^{SIM}$ consider both up-sharpness and down-sharpness, while $S^{ODIN}$ only considers up-sharpness.

**Sequential query stage** Moreover, we could also sequentially calculate up-sharpness and down-sharpness using the same input $x$ using the Sequential Composition Mode. Score $S^{SEQ}$ is defined as follows:

(C.13)
\begin{align*}& \begin{aligned} S^{SEQ}(\boldsymbol{x})&=\underset{j \in \mathcal{Y}}{max}\{ \underbrace{p_{j}(\boldsymbol{x})} _{magnitude}+ \underbrace{p_{j}(T_{plus}(\boldsymbol{x}))-p_{j}(\boldsymbol{x})}_{up-sharpness}\\&\quad+\underbrace{p_{j}(T_{plus}(\boldsymbol{x}))-p_{j}(T_{minus}(T_{plus}(\boldsymbol{x})))}_{down-sharpness}\}\\ &=\underset{j \in \mathcal{Y}}{max}\{ 2*p_{j}(T_{plus}(\boldsymbol{x}))- p_{j}(T_{minus}(T_{plus}(\boldsymbol{x})))\}.\\ \end{aligned}\end{align*}

In this mode, we sequentially calculate up-sharpness at the input x and down-sharpness at $T_{plus}(x)$.

### C.4 Score normalization

The score normalization is employed to resize the score obtained from the score function. The scores will become relatively small with the increase of hyperparameter $T$ proposed in Equation (C.4). Hence, we employ the following equations to normalize our scores:

(C.14)
\begin{align*} \begin{split} S_{refMax}&=\underset{\boldsymbol{x} \in \mathcal{D}_{ref}} {max}\ S(\boldsymbol{x}), \end{split}\end{align*}

(C.15)
\begin{align*} \begin{split} S^{norm}(\boldsymbol{x})&=\frac{S(x)-1/C^{ref}}{S_{refMax}-1/C^{ref}},\\ \end{split}\end{align*}

where $S_{refMax}$ is the max score obtained from the reference set via our proposed score functions; $S^{norm}(\boldsymbol{x})$ is the normalization score of input $\boldsymbol{x}$, which is within the range of $[0,1]$.

### C.5 Architecture and hyperparameters

We show the overall pipeline in Algorithm 1. We train a classifier $f$ with the reference set $\mathcal{D}_{ref}$ sampled from $\mathcal{P}_{typical}$. Model parameter $\theta $ is updated via backpropagation with the cross-entropy loss $\mathcal{L}_{CE}$ described in Equation (C.5). The number of neurons in the input layer is equal to the number of genes in the scRNA-seq dataset. The three subsequent hidden layers include 128, 64 and 32 units, respectively. The dropout rate of the dropout layer is 0.5 by default. We utilize AdamW [54] with $1e-4$ as the learning rate to optimize the network. The hyperparameter $T$ is set to be 1000, and the perturbation hyperparameter $\epsilon $ is 0.0014 (follow the parameter setting in [47]). The training batch size and iteration number are flexible for users, and we recommend users set the training batch size to at least 32 and the iteration number to at least 3000 to train the model well. All experiments are conducted on a GTX2080 Ti.

### C.6 Softeware comparison and settings

To evaluate the effectiveness of scNovel, we conducted a comparative analysis with various commonly employed techniques, including Scmap-cell, Scmap-cluster, scPred and scLearn. The evaluation criteria, as well as the input data, were obtained following the instructions and tutorials provided by each respective package. In order to maintain a level playing field, we adhered to the default parameters for all the methods utilized, including that of scNovel.

The software applications utilized in our study are as follows:

- scmap-Cell and scmap-Cluster, both of which were sourced from BioManager (https://git hub.com/hemberg-lab/scmap). The parameter settings for these functions were in accordance with the accompanying instructions.
- scPred, which was obtained from BiocManager (https://github.com/powellgenomicslab/scPred), was executed under default parameters. The algorithm selected the category with the highest score to generate the final result. It should be noted that scPred is better suited for processing relatively smaller datasets, as larger ones could require a considerable amount of time.
- scLearn sourced from Github (https://github.com/bm2-lab/scLearn) was also executed under the default parameter configuration.

## D. Appendix results

**Appendix Table 2:** Averaged experimental results among different datasets of scNovel and baseline methods. The $\color{red}{\mathrm{{red\ bold}}}$ and $\color{blue}{\mathrm{{blue\ bold}}}$ are the best result and second-best result, respectively, in corresponding evaluation metrics and $C^{novel}$

Evaluation	Methods	$C^{novel}$			
		1	2	3	4
FPR95(%)$\downarrow $	scmap-cluster [25]	55.85	54.56	42.17	$\color{blue}{\boldsymbol{{47.62}}}$
	scmap-cell [25]	68.74	70.23	79.44	73.84
	scPred [26]	60.43	65.09	73.86	66.96
	scLearn [27]	$\color{blue}{\boldsymbol{{40.74}}}$	$\color{blue}{\boldsymbol{{47.62}}}$	$\color{blue}{\boldsymbol{{39.29}}}$	54.52
	scNovel	$\color{red}{\boldsymbol{{22.82}}}$	$\color{red}{\boldsymbol{{21.31}}}$	$\color{red}{\boldsymbol{{28.74}}}$	$\color{red}{\boldsymbol{{25.95}}}$
**AUROC($\%$)$\uparrow $**	scmap-cluster [25]	80.78	78.38	83.56	$\color{blue}{\boldsymbol{{80.68}}}$
	scmap-cell [25]	84.01	77.60	81.65	80.63
	scPred [26]	78.45	73.48	76.63	70.49
	scLearn [27]	$\color{blue}{\boldsymbol{{84.30}}}$	$\color{blue}{\boldsymbol{{83.07}}}$	$\color{blue}{\boldsymbol{{87.45}}}$	77.61
	scNovel	$\color{red}{\boldsymbol{{96.33}}}$	$\color{red}{\boldsymbol{{94.29}}}$	$\color{red}{\boldsymbol{{94.64}}}$	$\color{red}{\boldsymbol{{94.16}}}$
AUPR(%)$\uparrow $	scmap-cluster [25]	$\color{blue}{\boldsymbol{{99.42}}}$	$\color{blue}{\boldsymbol{{98.59}}}$	97.62	$\color{blue}{\boldsymbol{{93.16}}}$
	scmap-cell [25]	99.30	97.23	95.44	91.27
	scPred [26]	99.24	97.85	94.08	86.00
	scLearn [27]	98.15	97.70	$\color{blue}{\boldsymbol{{97.71}}}$	91.60
	scNovel	$\color{red}{\boldsymbol{{99.78}}}$	$\color{red}{\boldsymbol{{99.41}}}$	$\color{red}{\boldsymbol{{98.70}}}$	$\color{red}{\boldsymbol{{97.72}}}$

**Appendix Table 3:** Experimental results with different score functions. The $\color{red}{\mathrm{{red\ bold}}}$ and $\color{blue}{\mathrm{{blue\ bold}}}$ are the best and second-best results, respectively, in corresponding evaluation metrics and $C^{novel}$

Evaluation	Methods	$C^{novel}$			
		1	2	3	4
FPR95(%)$\downarrow $	$S^{MSP}$	32.19	29.21	34.92	32.71
	$S^{ODIN}$	24.03	22.05	29.32	26.39
	$S^{SEQ}$	$\color{blue}{\boldsymbol{{22.94}}}$	$\color{red}{\boldsymbol{{20.99}}}$	$\color{blue}{\boldsymbol{{28.96}}}$	$\color{red}{\boldsymbol{{25.64}}}$
	$S^{SIM}$	$\color{red}{\boldsymbol{{22.82}}}$	$\color{blue}{\boldsymbol{{21.31}}}$	$\color{red}{\boldsymbol{{28.74}}}$	$\color{blue}{\boldsymbol{{25.95}}}$
**AUROC(%)$\uparrow $**	$S^{MSP}$	94.53	93.55	93.73	92.98
	$S^{ODIN}$	96.06	94.11	94.55	$\color{blue}{\boldsymbol{{93.94}}}$
	$S^{SEQ}$	$\color{blue}{\boldsymbol{{96.32}}}$	$\color{blue}{\boldsymbol{{94.26}}}$	$\color{blue}{\boldsymbol{{94.60}}}$	$\color{red}{\boldsymbol{{94.16}}}$
	$S^{SIM}$	$\color{red}{\boldsymbol{{96.33}}}$	$\color{red}{\boldsymbol{{94.29}}}$	$\color{red}{\boldsymbol{{94.64}}}$	$\color{red}{\boldsymbol{{94.16}}}$
AUPR(%)$\uparrow $	$S^{MSP}$	99.65	$\color{red}{\boldsymbol{{99.41}}}$	98.56	97.14
	$S^{ODIN}$	$\color{blue}{\boldsymbol{{99.75}}}$	$\color{red}{\boldsymbol{{99.41}}}$	98.66	97.48
	$S^{SEQ}$	$\color{red}{\boldsymbol{{99.78}}}$	$\color{red}{\boldsymbol{{99.41}}}$	$\color{blue}{\boldsymbol{{98.69}}}$	$\color{blue}{\boldsymbol{{97.71}}}$
	$S^{SIM}$	$\color{red}{\boldsymbol{{99.78}}}$	$\color{red}{\boldsymbol{{99.41}}}$	$\color{red}{\boldsymbol{{98.70}}}$	$\color{red}{\boldsymbol{{97.72}}}$

**Appendix Table 4:** Experimental results with different neural network architecture, including removing BN (scNovel w/o BN) and removing ELU activation function (scNovel w/o BN). The $\color{red}{\mathrm{{red\ bold}}}$ and $\color{blue}{\mathrm{{blue\ bold}}}$ are the best and second-best results, respectively, in corresponding evaluation metrics and $C^{novel}$. The experiment results suggest that both BN and ELU are useful to boost performance

Evaluation	Methods	$C^{novel}$			
		1	2	3	4
FPR95(%)$\downarrow $	scNovel w/o BN	$\color{blue}{\boldsymbol{{24.28}}}$	26.63	$\color{red}{\boldsymbol{{23.32}}}$	$\color{blue}{\boldsymbol{{30.17}}}$
	scNovel w/o ELU	26.44	$\color{blue}{\boldsymbol{{22.88}}}$	31.13	31.63
	scNovel	$\color{red}{\boldsymbol{{22.82}}}$	$\color{red}{\boldsymbol{{21.31}}}$	$\color{blue}{\boldsymbol{{28.74}}}$	$\color{red}{\boldsymbol{{25.95}}}$
**AUROC(%)$\uparrow $**	scNovel w/o BN	94.85	89.15	93.42	91.58
	scNovel w/o ELU	$\color{blue}{\boldsymbol{{96.03}}}$	$\color{blue}{\boldsymbol{{93.47}}}$	$\color{blue}{\boldsymbol{{94.24}}}$	$\color{blue}{\boldsymbol{{93.01}}}$
	scNovel	$\color{red}{\boldsymbol{{96.33}}}$	$\color{red}{\boldsymbol{{94.29}}}$	$\color{red}{\boldsymbol{{94.64}}}$	$\color{red}{\boldsymbol{{94.16}}}$
AUPR(%)$\uparrow $	scNovel w/o BN	99.74	99.09	98.46	95.88
	scNovel w/o ELU	$\color{blue}{\boldsymbol{{99.77}}}$	$\color{blue}{\boldsymbol{{99.35}}}$	$\color{blue}{\boldsymbol{{98.56}}}$	$\color{blue}{\boldsymbol{{97.03}}}$
	scNovel	$\color{red}{\boldsymbol{{99.78}}}$	$\color{red}{\boldsymbol{{99.41}}}$	$\color{red}{\boldsymbol{{98.70}}}$	$\color{red}{\boldsymbol{{97.72}}}$

## E. Discussion of scNovel and scBalance

The recent work named scBalance [16] focuses on identifying rare cell types in large datasets, while we focus on novel rare cell detection. We explain it in detail below. The scBalance specializes in classifying rare cells into known categories within single-cell sequencing datasets, unable to identify previously unknown cell types. In contrast, scNovel is aimed at detecting novel rare cell types by determining if a cell type has been observed before, thus capable of identifying new cell types beyond mere classification. The main limitation of scBalance is its inability to classify cells outside its trained categories, leading to potential misclassification. scNovel addresses this by detecting cells that don’t fit into known categories, improving accuracy in identifying novel cell types. However, scNovel could be enhanced with a more robust architecture and refined scoring function to better distinguish between typical and novel cell distributions, indicating areas for future improvement in both models. We employ the same network architecture like scBalance and loss function, while we propose new score functions ($S^{ODIN}$, $S^{SEQ}$ and $S^{SIM}$) to further improve performance. Therefore, if we directly apply scBalance for novel rare cell detection, the performance will be degraded to the performance of SMSP. Therefore, the experiment comparison between scNovel and scBalance is shown in Appendix Table 3, while $S^{MSP}$ is the performance of scBalance.
